# Involvement of Glutathione Depletion in Selective Cytotoxicity of Oridonin to p53-Mutant Esophageal Squamous Carcinoma Cells

**DOI:** 10.3389/fonc.2019.01525

**Published:** 2020-01-15

**Authors:** Yinchao Li, Nana Li, Jianxiang Shi, Tanzeel Ahmed, Hongmin Liu, Jiancheng Guo, Wenxue Tang, Yongjun Guo, Qi Zhang

**Affiliations:** ^1^Key Laboratory of Technology of Drug Preparation, Ministry of Education of China, Zhengzhou, China; ^2^School of Pharmaceutical Sciences, Zhengzhou University, Zhengzhou, China; ^3^Center for Precision Medicine, Zhengzhou University, Zhengzhou, China; ^4^The Affiliated Cancer Hospital of Zhengzhou University, Zhengzhou, China

**Keywords:** oridonin, RNA seq, glutathione, esophageal squamous cell carcinoma cells, p53-mutation

## Abstract

Oridonin, a diterpenoid compound isolated from traditional Chinese medicine Rabdosia rubescens, has shown antitumor effects to esophageal cancer. However, its molecular mechanism is not fully understood, which limits its clinical application. In the present study, we used RNA-seq analysis to check the transcriptome changes after oridonin treatment and we found genes controlling the GSH-ROS system were up-regulated, namely *SLC7A11, TXNRD1, TRIM16, SRXN1, GCLM*, and *GCLC*. Furthermore, our data suggest that oridonin significantly increased the production of ROS in EC109 and TE1 cells, which can be inhibited by NAC. Interestingly, oridonin can dramatically reduce intracellular GSH levels in TE1 cells in a concentration and time-dependent manner. In addition, cell death caused by oridonin was strongly inhibited by GSH (1 mM), while GSSG (1 mM) had little effect. At the same time, we also found that oridonin showed selective cytotoxicity to esophageal squamous carcinoma cell with p53 mutation since mut-p53 cells had lower *SLC7A11* expression, a component of the cystine/glutamate antiporter. We also found that γ-glutamyl cysteine synthetase inhibitor (BSO) synergizes with oridonin to strongly inhibit EC109 cells at a low dose. These results suggested that the antitumor effects of oridonin are based on its –SH reactivity and glutathione depletion. Esophageal squamous carcinoma cells with p53-mutation showed hypersensitivity to oridonin because of the suppression of SLC7A11 expression by p53 mutation.

## Introduction

Esophageal cancer is a common malignant tumor of the digestive system. It is mainly divided into esophageal adenocarcinoma (EAC) and esophageal squamous cell carcinoma (ESCC) according to different etiology and pathological features (1–3). However, due to frequent recurrence after surgery, the effect is not satisfactory after chemotherapy or radiotherapy, their overall 5-year survival rate is <20% (4, 5). Therefore, the development of new therapeutic drugs or the search for new therapeutic targets is very urgent for the clinical treatment of esophageal cancer.

Oridonin, an active diterpenoid compound extracted and purified from Rabdosia rubescens, has great effects on suppressing the proliferation of esophageal, gastric, liver, and colon cancer cells (6–8). Previous shreds of evidence have shown that oridonin-mediated apoptosis involves multiple proteins and pathways, such as protein kinase B (AKT), the nuclear factor-kappa B (NF-κB) and mitochondrial redox signaling pathway (9–11). However, the molecular target of the anti-tumor effect of oridonin has not been fully elucidated, which limits the further clinical application of oridonin.

In this study, we used a full transcriptome analysis technique (RNA-seq) to elucidate the potential molecular targets of oridonin. We found that the expression of heat shock-related protein, such as *HSPA1A, HSPA1B, BAG3, HSPH1*, and *DNAJB1* was significantly increased after treatment with oridonin. These results validated that HSP70 is one of the molecular targets for the action of oridonin, which is in keeping with the reported by Vasaturo et al. (12). Furthermore, *TRIM16, SLC7A11, TXNRD1, SRXN1, GCLM, GCLC*, and other genes are closely related to intracellular GSH, ROS levels were also upregulated. According to relevant literature reports (13), we found that these gene expression changes were highly consistent with changes in gene expression after APR-246 treatment of cells, which mainly consumes intracellular glutathione by binding to -SH on GSH in cancer cells, eventually causing the cell death via excessive lipid peroxidation (13). Through chemical structure analysis, we found that APR-246 has similar reactive groups to oridonin (14). To this end, we further verified that oridonin has the similar function of targeting GSH.

## Methods

### Reagents

Oridonin (B20310) was purchased from Yuanye company (Shanghai, China. APR-246 was provided by MCE (USA). GSH and GSSG Assay Kit (S0053) and Reactive Oxygen Species Assay Kit (S0033) were from Beyotime. Annexin V-FITC Apoptosis Detection Kit (KGA106) was from KeyGEN. Lipofectamine 3000 Reagent (L3000008) was from Invitrogen. Anti-xCT (ab175186) and GAPDH Monoclonal Antibody (AC033) were supplied by Abcam and Abclonal, respectively. Goat Anti-Mouse IgG (H + L)/HRP antibody (bs-40296G-HRP) was from Bioss.

### Cell Cultures

The following panel of human esophageal cancer cell lines was used; TE-1, KYSE-30, and KYSE-450 (mut-p53); EC109, KYSE70, and KYSE 410 (wt-p53). KYSE-450 and EC109 cells were cultured in Dulbecco modified Eagle medium (DMEM). TE-1, KYSE-30, KYSE70, and KYSE410 cells were maintained in RPMI 1640 medium. All culture media supplemented with 10% FBS, 100 U ml^−1^ penicillin and 100 mg/ml streptomycin. Otherwise, all cells were cultured at 37°C with 5% CO_2_.

### Cell Viability Assay

The 3-(4,5-dimethylthiazol-2-yl)-2,5-diphenyl-tetrazolium bromide (MTT) was used to detect cytotoxicity. EC109, KYSE70, KYSE410, TE-1, KYSE-450, and KYSE-30 cells were seed at a density of 5 × 10^3^ per well in 96-well plates. The following day, the cells were treated with oridonin for 24, 48, and 72 h respectively. And then MTT (5 mg/ml) was added and incubated for 4 h at 37°. Measurement of absorbance at 562 nm is done by microplate reader (Thermo).

### Apoptosis Detection

Apoptosis of EC109 and TE1 cells was assessed by flow cytometry. EC109 and TE1 cells were plated in 6-well plates at a density of 5 × 10^5^ per well. The next day, cells were treated with different concentrations of oridonin for 24 h, stained with Annexin V-FITC Apoptosis Detection Kit (KeyGEN) according to the manufacturer's instructions, and apoptosis rate was measured by flow cytometry (BD).

### RNA Sequencing

TE-1 and EC109 cells were treated with 30 μM of oridonin or DMSO control for 4 h. Total RNA from three duplicate wells per group were extracted by using RNA prep Pure Cell/Bacteria Kit (Qiagen) according to the manufacturer's instructions. The RNA concentration and integrity were detected by Nandrop (Thermo) and Agilent 2100 Bioanalyzer (Agilent Systems. RNA-seq cDNA libraries were prepared using TruSeq Stranded mRNA LT Sample Prep Kit (Illumina). The sequencing process was completed by Microanaly Technology Co., Ltd., Shanghai. The paired-end library was sequenced with the Illumina HiSeq 4000 with a read length of 2 × 150 bp.

### Analysis of RNA-seq Data

The RNA-seq data analysis protocol followed the best practices proposed by Conesa et al. (15). In brief, Fast QC was used to do quality control checks for raw sequencing data. STAR aligner was used to align reads to the Ensembl human reference genome (GRCh37, version 75) (16). Adapter trimming was not performed since the STAR aligner can soft clip the ends of reads and handle contaminant sequences at the ends properly. Salmon was used to in quantitating the expression of genes (17). All the mentioned software was implemented in the bcbio-nextgen analysis pipeline and the pipeline was used to analyze RNA-seq data. Then bcbioRNASeq R package was used to conduct differential gene expression analysis.

### Gene Expression With Quantitative RT-PCR

RNA from cell lines was isolated using the RNA prep Pure Cell / Bacteria Kit (Tiangen), and first-strand cDNA was prepared with PrimeScript™ RT Master Mix (TaKaRa). Real-time PCR was performed using the ChamQ™ Universal SYBR qPCR Master Mix (Vazyme). Gene expression was normalized to GAPDH. And data is analyzed using the 2^−ΔΔct^ method.

### Intracellular Glutathione and ROS Assay

TE-1 and EC109 cells were a seed to 6-well plates in triplicates and treated. Intracellular total glutathione content is assayed by GSH and GSSG Assay Kit (S0053, Beyotime) according to the manufacturer's instructions. The total GSH content was calculated by the standard curve. Intracellular ROS were detected using Reactive Oxygen Species Assay Kit (S0033, Beyotime. Cells were incubated for 20 min at 37°C in the dark with 10 μM DCFH-DA, collected and analyzed using BD Accuri™ C6 Plus Flow Cytometer. The data were analyzed through the Flowjo software.

### Western Blot Analysis

Cells were lysed in RIPA buffer (solarbio) containing 1 mM PMSF (solarbio) for 30 min at 4°C. After centrifugation at 13,000 × g at 4°C for 30 min, the supernatant fractions were denatured in SDS for 10 min and quantified by BCA Protein Assay Kit (P0012S, Beyotime). Equal amounts of protein were separated by 10% SDS-PAGE and transferred to polyvinylidene difluoride membranes. Membranes were blocked in Blocking Buffe (P0023B, Beyotime) for 1 h at room temperature, and then incubated overnight with primary antibodies at 4°C. Blots were washed by 1 × PBST buffer thrice (5 min each), and then incubation with the Goat Anti-Mouse IgG (H + L)/HRP antibody (bs-40296G-HRP) for 1 h at room temperature. Blots were visualized by using BeyoECL Moon (P0018FS, Beyotime). Protein quantitative analysis was calculated using ImageJ software.

### siRNA Knockdown

SLC7A11 siRNA Transfection cells were seed in 6-well plates and transfected with SLC7A11 siRNA (RIBOBIO, China) and control siRNA using Lipofectamine™ 3000 Reagent (L300008, Invitrogen) according to the manufacturer's instructions. After transfection for 72 h, cells were harvested for further experiments. The efficiency of siRNA knockdown was determined by RT-PCR and Western blot analysis.

### Statistical Analysis

All data were expressed in mean ± SD and performed at least three independent experiments. Significant differences between groups were assessed by one-way ANOVA (^*^P < 0.05; ^**^P < 0.001).

## Result

### Oridonin Induces Apoptosis in TE1 and EC109 Cells

**Figure 1:**
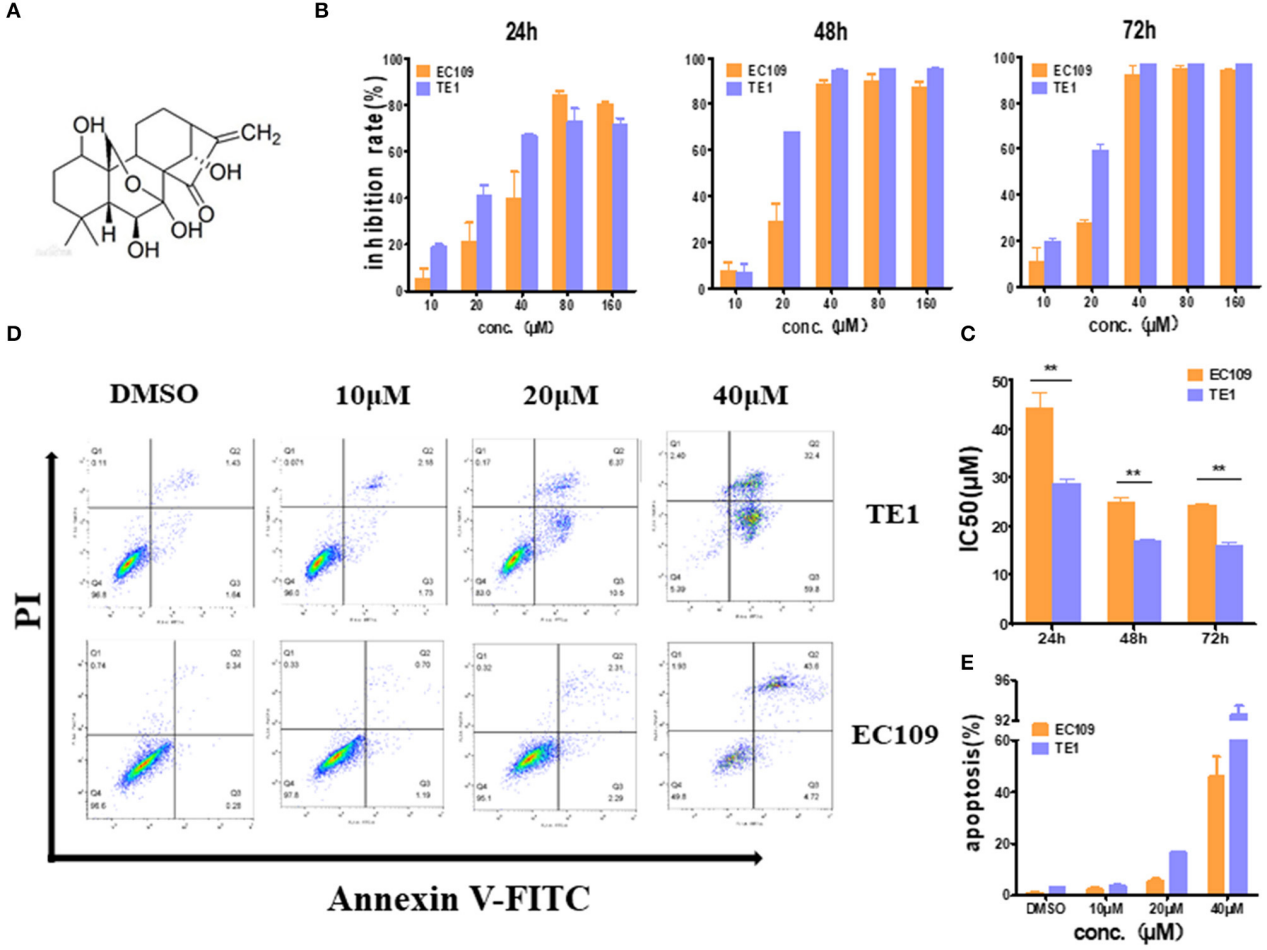
Oridonin induces apoptosis in esophageal cancer cell lines TE1 and EC109. **(A)** Chemical structure of oridonin. **(B)** EC109 and TE-1 cells were treated with different concentrations of oridonin or vehicle for 24, 48, and 72 h. The cell inhibition rate was determined using MTT assay, **(C)** IC50 values comparison between EC109 and TE-1 cells. **(D)** Fluorescence-activated cell sorting plots of Annexin V/PI-labeled cells treated with 10, 20, and 40 μM oridonin for 24 h. **(E)** The apoptosis rate is expressed as the mean ± SD (*n* = 3). The data were obtained from three independent experiments. *P < 0.05, **P < 0.01 compared to EC109 cell group.

To confirm the cytotoxicity of oridonin to EC109 cells and TE1 cells, MTT assay was employed to detect the cell viability with or without oridonin treatment (Figure 1A). Oridonin showed effective cytotoxicity on EC109 and TE-1 cells in a time- and dose-dependent manner (Figure 1B). However, TE-1 cells were more sensitive than EC109 cells to oridonin, and the IC50 values of TE-1 cells were significantly reduced regardless of the length of treatment, compared with EC109 cells (Figure 1C, *P* < *0.01*). Our flow cytometry analysis also indicated that oridonin increases the percentage of apototic (annexin V-PI positive) cells in TE1 compared to EC109 cells, especially when treated with 40 μM oridonin (*P* < *0.01*, Figures 1D,E).

### Effect of Oridonin on Global Gene Expression

The molecular target of Oridonin is not fully elucidated. Transcriptome analysis might be a convenient way to explore its potential molecular mechanism. Here we treated the cells for 4 h with 30 μM oridonin, a dosage very close to the IC50 values of oridonin in TE1 and EC109 cells. Then total RNA was extracted and proceed to do RNA-seq analysis.

**Figure 2:**
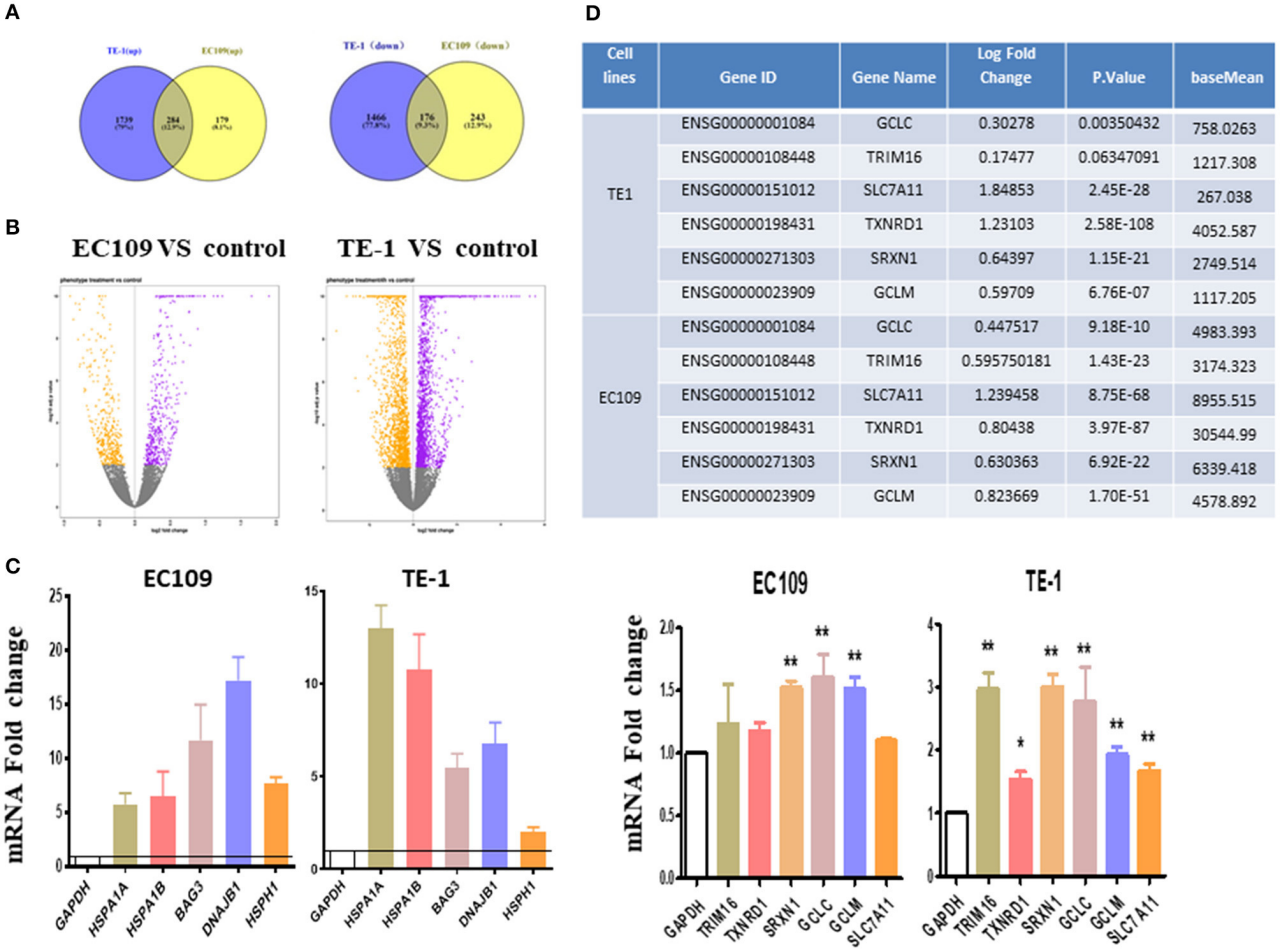
Transcriptome changes induced by oridonin using RNA-seq. **(A)** Venn diagram showing amounts of differentially expressed genes after treatment with oridonin for 4 h in EC109 cells and TE1 cells. (≥two-fold up/down-regulated, *P* < 0.01). **(B)** The “volcano plot” graph of the differentially expressed genes in EC109 and TE1 cells. **(C)** Verify RNAseq by qPCR analysis. TRIM16, TXNRD1, SRXN1, SLC7A11, GCLC, or GCLM and HSPA1A, HSPA1B, BAG3, DNAJB1, HSPH1 in EC109 cells and TE1 cells, respectively. Cells were treated with 30 μM oridonin or DMSO for 4 h. **(D)** Differentially expressed genes related to GSH in RNAseq. *P < 0.05, **P < 0.01 compared to DMSO group.

Differentially expressed genes (DEGs) were analyzed using DESeq2 (adjusted *P*-value < 0.01, log2 |FC| 0). In general, the number of DEGs uniquely present in TE1 cells after treatment was 2,023 (up-regulated) and 1,642 (down-regulated), while 463 (up-regulated) and 419 (down-regulated) genes were significantly present in EC109 cells (Figures 2A,B, *P* < 0.01). Supplementary Tables 1, 2 list the top 20 most upregulated and downregulated genes of TE1 and EC109 cells, by fold change. We verified the differentially expressed genes by RT-PCR and found that protein misfolding stress responsive genes, such as *HSPA1A, HSPA1B, BAG3, HSPH1*, and *DNAJB1* were significantly up-regulated (Figure 2C), which demonstrates that oridonin cause genes upregulation similar to heat shock response (12). In addition, genes related to the regulation of reactive oxygen species and glutathione activity, such as *TRIM16, SLC7A11, TXNRD1, SRXN1, GCLM*, and *GCLC* are significantly enhanced in TE1 cells (Figure 2D). The upregulation of SLC7A11 expression was shown by RNA-seq, but not confirmed by RT-PCR in all cell lines. Both *TXNRD1* encoding thioredoxin reductase (TrxR) and *SRXN1* encoding thiourea reductase are important intracellular redox regulators (13). The change of intracellular glutathione content is controlled by the cystine-glutamate reverse transporter, which was encoded by *SLC7A11* (14). The *GCLC* and *GCLM* genes mainly encode γ-glutamyl cysteine synthetase, which is a rate limiting enzyme for the synthesis of GSH (18). Therefore, the increased expression upregulation of these genes may also be interpreted as a response to increased intracellular ROS. Although *TRIM16* has not been previously reported to be implicated in controlling redox balance, it was regulated by another drug, APR-246, which regulates intracellular reactive oxygen species and GSH content (13). Therefore, we next explored the effects of oridonin on intracellular ROS and GSH. To further demonstrate the similar molecular mechanism between APR-246 and oridonin, we use APR-246 as a comparison in the following studies.

### Oridonin Promotes Intracellular ROS Accumulation

**Figure 3:**
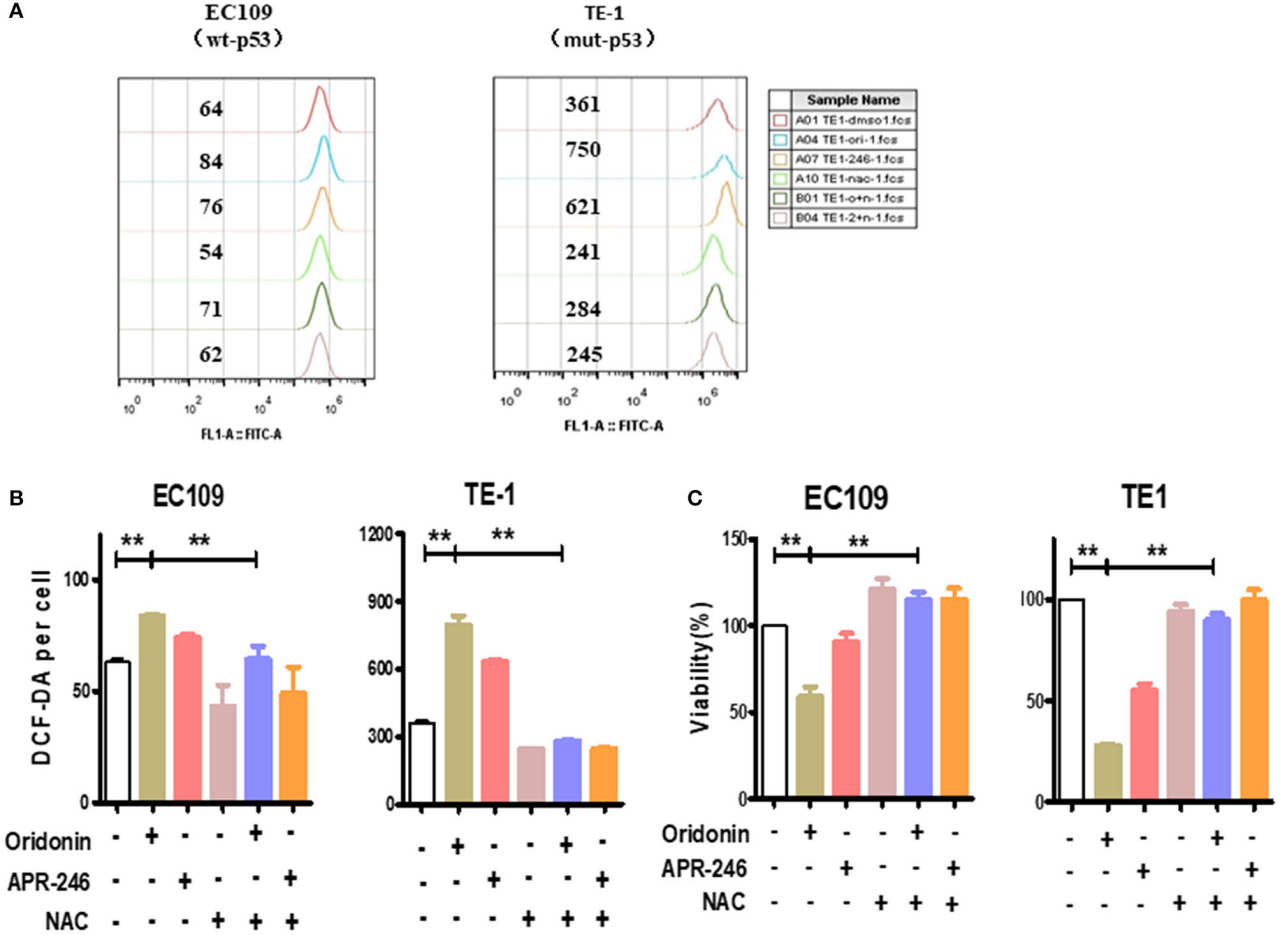
ROS produced by the oridonin treatment. **(A)** ROS analysis was treated with 30 μM oridonin, 30 μM APR-246 and/or 5 mM NAC in TE1 cells, and treated with 40 μM oridonin, 30 μM APR-246 and/or 5 mM NAC in EC109 cells for 4 h. The levels of intracellular ROS were determined using DCF-DA. **(B)** The mean fluorescence intensity is shown. **(C)** Viability at 24 h post-treatment with 30 μM oridonin (TE1 cells) or 40 μM oridonin (EC109 cells), 30 μM APR-246 and/or 5 mM NAC. All data are presented as the mean ± SD, **P < 0.01 compared to the DMSO.

To investigate whether the cytotoxicity of oridonin is related to reactive oxygen species (ROS), we measured intracellular ROS levels by flow cytometry. Of interest, we found that the baseline level of ROS in TE1 cells is much higher compared to EC109 cells (Figures 3A,B) While oridonin (30 and 40 μM) significantly induced intracellular ROS generation at 4 h for both cell lines (*P* < 0.01), N-acetyl-L-cysteine (NAC), Sulfhydryl-containing antioxidant, can significantly inhibit the ROS production induced by oridonin in EC109 cells and TE-1 cells (Figures 3A,B). In addition, NAC (5 mM) can also obviously reverse the cytotoxicity caused by oridonin in EC109 cells and TE-1 cells (Figure 3C). It is well known that excessive accumulation of reactive oxygen species leads to oxidative damage and cell death. Therefore, we speculated that the reason why TE1 cells retains higher sensitivity to oridonin could be its higher level of endogenous ROS, thus additional oxidative damage caused byoridonin would easily bring the cellular oxidative damage level over the given lethal threshold.

### Oridonin Reduces Intracellular GSH Level

**Figure 4:**
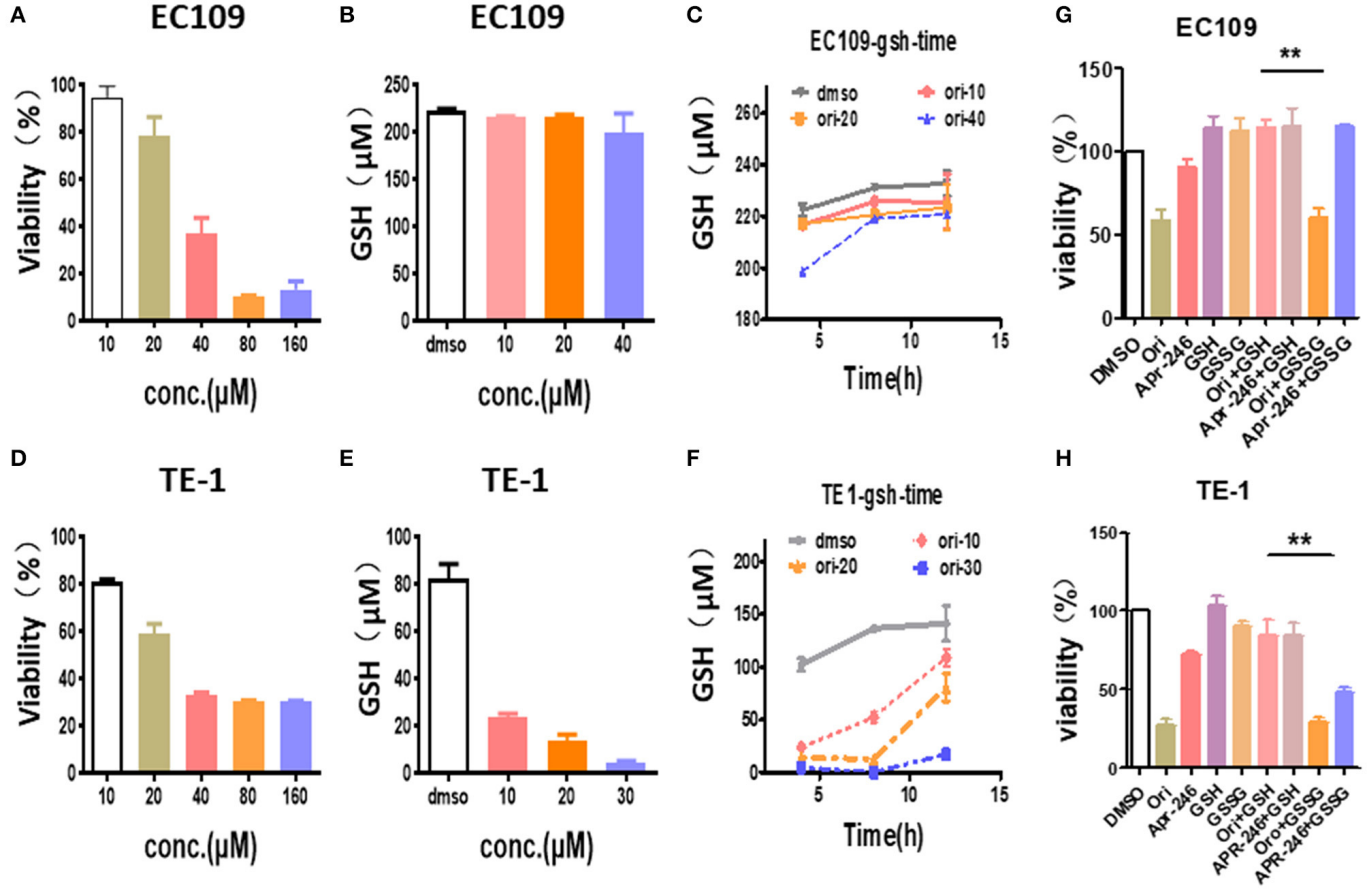
Oridonin induced cytotoxicity by depleting intracellular GSH. **(A,C)** Viability **(A)**, intracellular GSH content **(B,C)** were determined treated with oridonin in EC109 cells. **(D-F)** Viability **(D)**, intracellular GSH content **(E,F)** were determined treated with oridonin in TE1 cells. **(G-H)** Viability at 24 h post-treatment with 1 mM GSH, 1 mM GSSG and/or 40 μM oridonin (EC109 cell) **(G)** or 30 μM oridonin (TE1 cell) **(H)**. **P < 0.01, compared to treatment either of the compounds alone.

We explored the effects of oridonin on GSH in esophageal cancer cells. Our results showed that the treatment of TE1 cells and EC109 cells with different doses of oridonin results in lower intracellular GSH (Figures 4A,B,D,E). Among them, oridonin rapidly reduced GSH levels in a concentration and time-dependent manner in TE1 cells, however, GSH levels in EC109 cells were only slightly reduced at the median lethal dose (Figures 4C,F). These results also confirmed a significantly different endogenous ROS status comparing with TE1 cells and EC109 cells: in TE1 cells the higher baseline level of oxidative stress has consumed a significant portion of cellular GSH reservoir, thus oridonin dependent GSH depletion would be more apparent in such context.

In addition, to further confirm that oridonin-induced cell death is mediated through GSH depletion, we explored the effects of GSH and GSSG (oxidized glutathione) on cell viability by MTT assay (Figures 4G,H). Our results indicated that cell death caused by oridonin was strongly inhibited by GSH (1 mM) in both EC109 and TE1 cells, while GSSG had little effect. This suggests that the sulfhydryl group in the GSH structure may be the critical active group contracting oridonin.

### *SLC7A11* Blockade Causes Esophageal Cancer Cells to Be More Sensitive to Oridonin

**Figure 5:**
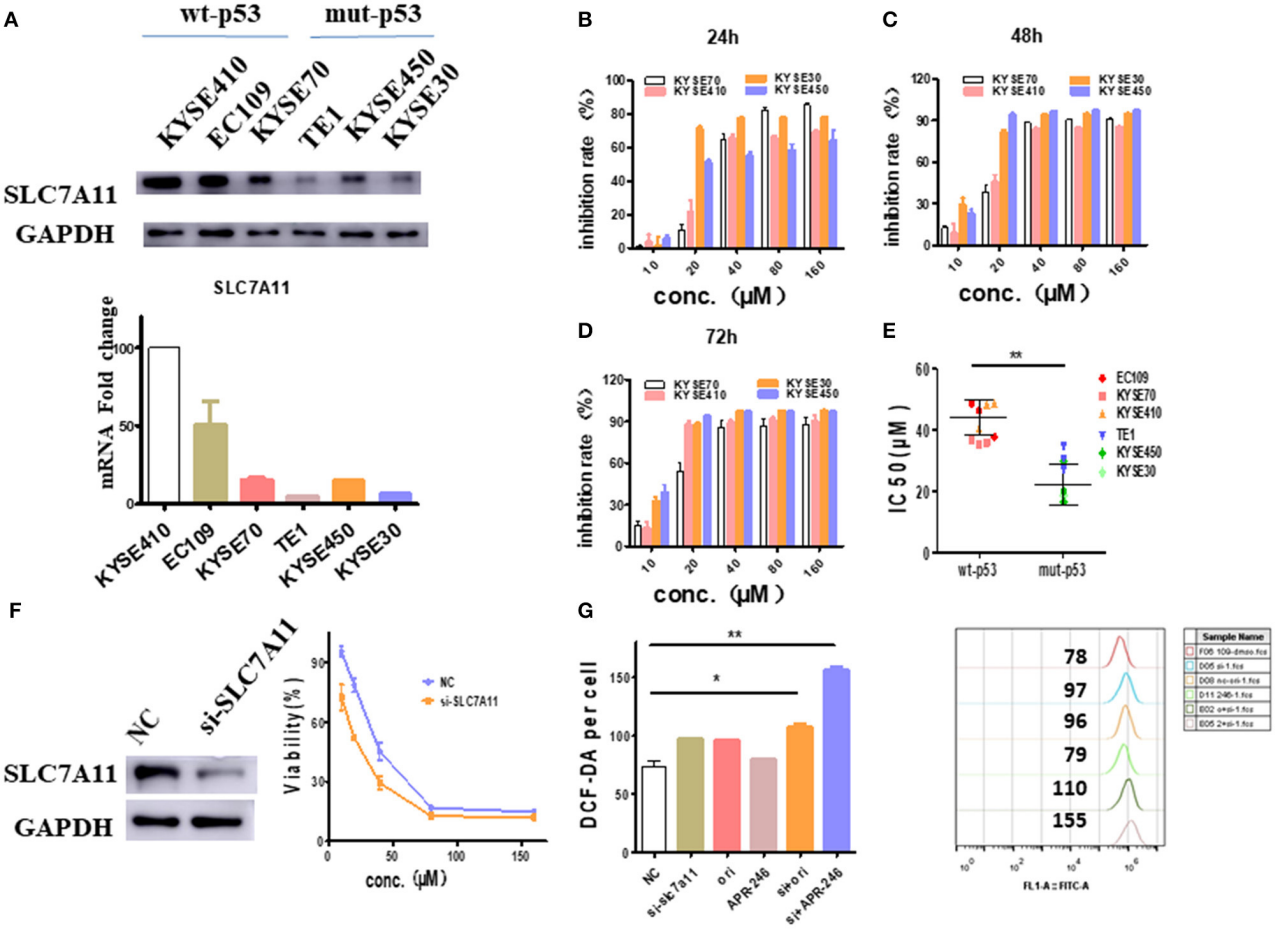
*SLC7A11* expression predicts and affects ESCC sensitivity to oridonin. **(A)** Transcriptional expression of the *SLC7A11* gene in esophageal squamous carcinoma cells. **(B–D)** KYSE70, KYSE410. KYSE30 and KYSE450 cells were treated with different concentrations of oridonin or vehicle for 24 h **(B)**, 48 h **(C)**, and 72 h **(D)**. The cell's inhibition rate was determined using MTT assay, **(E)** IC50 values comparison for them. **(F)** Oridonin was applied 72 h post-transfection of SLC7A11 and non-targeting control (NC) siRNA with viability measured at 24 h post oridonin by MTT assay, **(G)** ROS analysis in cells treated with 40 μM oridonin 72 h after NC or *siSLC7A11* transfection. Intracellular ROS was detected after treatment with oridonin for 4 h. *P < 0.05, **P < 0.01, compared to the NC.

Since the intracellular GSH is regulated by *SLC7A11*, a cysteine/glutamate antiporter (14), the relationship between *SLC7A11* and oridonin cytotoxicity needs to be further demonstrated. We first verified the expression of *SLC7A11* in multiple esophageal squamous carcinoma cells by RT-PCR and western-blot. We found that SLC7A11, transcript and protein levels are significantly lower in cell lines harboring mutant p53 mutations (Figure 5A). This result is consistent with the previous findings of David et al., that mutant p53 can repress the expression of *SLC7A11* (14). Furthermore, we detected multiple esophageal squamous carcinoma cell lines (with either wild type p53 or mutant p53) by MTT assay. Consistent with intracellular SLC7A11 expression, KYSE450, and KYSE30 cells have significantly lower IC50 values than KYSE70, KYSE410 cells (Figures 5B–E). Our data suggest that mut-p53 esophageal cancer cells are more sensitive to oridonin, probably due to depletion of GSH and systemic xC blockade causing cysteine deficiency, which leads to impaired GSH synthesis. To examine whether such differential oridonin sensitivity is indeed mediated by mutant p53 dependent *SLC7A11* downregulation, we investigate whether the sensitivity of wt-p53 esophageal cancer cells to oridonin can be enhanced by knocking down the *SLC7A11* gene. Indeed, we found that knocking down *SLC7A11* (Figure 5F) significantly increased the cytotoxicity of oridonin to EC109 cells, and resulted in increased ROS levels correspondingly (Figure 5G).

### Inhibition of GSH Synthetase Synergizes With Oridonin to Inhibit EC109 Cells

**Figure 6:**
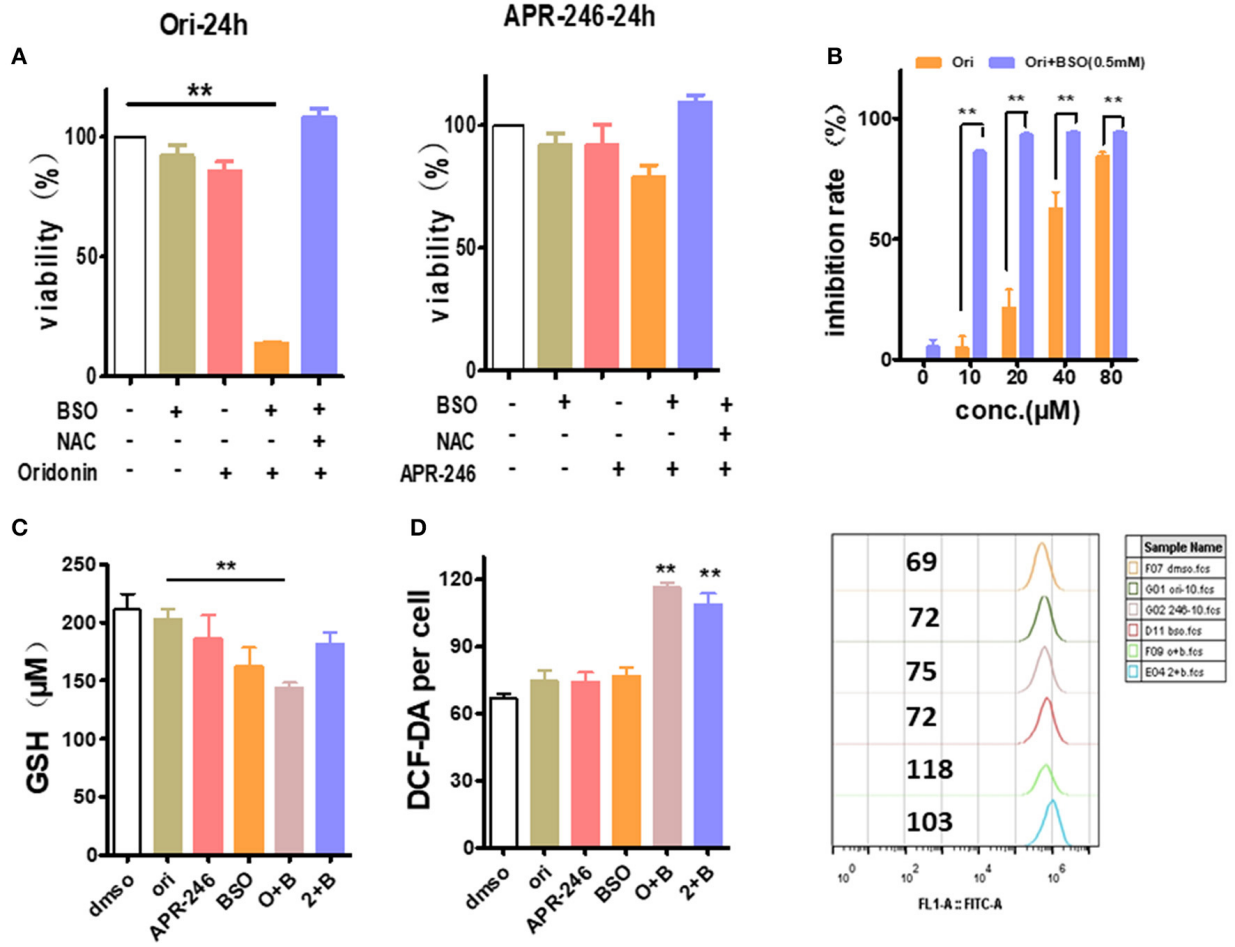
Enhancement of oridonin cytotoxicity with GSH synthesis inhibitor BSO. **(A)** EC109 cells were incubated with BSO (0.5 mM) or/and oridonin (10 μM) or APR-246 (10 μM) for 24 h. and cell viability was assessed using MTT. **(B)** Increased doses of oridonin enhance synergy with BSO. EC109 cells were incubated for 24 h with serial concentrations of oridonin in the presence of 0.5 mM BSO. **(C)** GSH analysis of EC109 cells treated with 10 μM oridonin or 10 μM APR-246 and/or 0.5 mM BSO. **(D)** ROS of EC109 cells were detected at 4 h post-treatment with oridonin (10 μM) or APR-246 (10 μM) and/or 0.5 mM BSO. **P < 0.01, compared to treatment with vehicle control or either of the compounds alone.

Besides *SLC7A11* or similar glutamate transporter, the intracellular GSH content is also greatly affected by γ-glutamyl cysteine synthetase, a rate limiting enzyme of GSH synthesis (19). Therefore, we used L-Buthionine-sulfoximine (BSO), γ-glutamyl cysteine synthetase inhibitor, to examine whether it synergizes with oridonin to eliminate EC109 cells. At sub IC50 dosage, neither oridonin (10 μM) nor BSO (0.5 mM) induced any significant cell cytotoxicity (mean values of 5.1 and 5.6%, respectively) in EC109 cells, but they had a striking synergistic cytotoxic effect in combination, which can be reversed by NAC, suggesting the synergy is through increasing cellular ROS stress. In addition, their synergistic effect remains despite gradual increase of with oridonin dosage (Figures 6A,B).

Next, to assess whether the cytotoxicity caused by the combination of oridonin and BSO is related to intracellular GSH and ROS levels in EC109 cells. As shown in Figure 6C, Oridonin alone did not lower intracellular GSH at a concentration of 10 mM. However, when combined with BSO, a significant reduction of intracellular GSH was observed in EC109 cells. At the same time, we found an increase of intracellular ROS levels in EC109 cells specifically with the synergistic treatment (Figure 6D). This suggests that in the case of BSO combined with oridonin, the increase of intracellular GSH consumption may be responsible for the increased ROS production, indicated the role of glutathione depletion in BSO plus oridonin-induced ROS production and cell death.

## Discussion

The current chemotherapy for esophageal squamous cancer is very limited and there is still an urgent need to find new chemicals for ESCC. Oridonin, a major diterpenoid component of leaf extracts from Rabdosia rubescens, has been demonstrated to be effective to a variety of cancers, such as esophageal, leukemia, lung, pancreatic, prostate, breast, and colon both *in vivo*/*vitro* (20). Although great efforts have been devoted to study the molecular mechanism of its anti-cancer effects, there is still a lack of concrete evidence to fully elucidate at the whole transcriptome level. In the present study, we employed RNA-seq for qualitative and quantitative analysis of all transcriptome changes induced by oridonin. Firstly, we chose EC109 and TE1, two commonly used ESCC cancer lines to explore the potential molecular targets of oridonin. We verified that oridonin induces apoptosis in both TE1 and EC109 cells. To see the early transcriptome changes induced by oridonin at a cytotoxic dose, we chose 30 μM of oridonin and 4 h treatment and then extracted all the RNAs to do RNA-seq analysis. By DESeq2 analysis, we found 284 genes were up-regulated in two cell lines, and 176 genes were down-regulated in both cells.

By further analyzing the function of those up-regulated genes, we believe that at least two sets of genes played major roles in oridonin cytotoxicity to ESCC cells. The first set of genes are heat shock genes, such as *HSPA1A, HSPA1B, BAG3, HSPH1, DNAJB1, HSPA8, HSP90AA1, DNAJA4*, and *DNAJA1*, whose transcriptions are strongly regulated by HSF1. HSF1 binds to HSP70 and loses its transcriptional activity. When oridonin binds to its targets, HSF1 may dissociate with HSP70 and therefore is activated, thereby increasing the expression of downstream heat shock proteins (21, 22). Those results prove theoretically that HSP70 is one of the molecular targets. The second set of genes are *TRIM16, SLC7A11, TXNRD1, SRXN1, GCLM*, and *GCLC*, etc., which are also closely related to intracellular ROS and GSH levels (23). Through literature research (13), we found that these genes are highly consistent with the gene profile of the APR-246, so we speculated that the anti-tumor mechanism of oridonin may be similar to APR-246, inducing tumor cell death by depleting intracellular GSH (14, 24, 25). The next, we treated EC109 cells and TE1 cells with oridonin or APR-246, respectively, and tested intracellular ROS and GSH levels. To further verified that GSH played a major role in oridonin cytotoxicity to ESCC cells, we designed several experiments to test this hypothesis. First, we found that TE1 cells had a higher level of ROS compared to EC109 cells, both oridonin and APR-246 can increase intracellular ROS levels, which can be reversed by NAC. It is well-known that excessive accumulation leads to oxidative damage and cell death (11). Therefore, we made hypothesis that esophageal cancer cells with elevated ROS are sensitive to further oxidative damage caused by oridonin, and thus are more likely to induce cell death. On the other hand, we found that oridonin can rapidly reduce GSH levels in TE1 cells in a time- and dose-dependent manner, but less affects EC109 cells. In addition, GSH can significantly resist the cytotoxicity of oridonin and APR-246 in both cell lines by MTT assay, but GSSG cannot. These results further confirm that the target of oridonin is consistent with APR-246, which is combined with the thiol group of GSH (26). For EC109 cells with high GSH content, oridonin plays a toxic role mainly by binding to other thiol-containing proteins in addition to GSH, such as heat shock proteins, while for TE1 cells with less GSH content, oridonin induces apoptosis mainly by depleting intracellular GSH. Second, we test if the expression of *SLC7A11*, cystine/glutamate antiporter and essential for GSH synthesis, is related to the sensitivity of oridonin, we examined the expression of *SLC7A11* in multiple esophageal squamous carcinoma cell lines and their cytotoxicity against oridonin. Our data showed that cell lines with higher expression of *SLC7A11*, such as EC109, KYSE70, and KYSE410 treated with oridonin, respectively, had lower IC50 values after 24 h than cells with lower expression of *SLC7A11*, such as KYSE450, KYSE30 and TE1 cells. In fact, *SLC7A11* expression is down-regulated by mut-p53. Since KYSE450, KYSE30 and TE1 cells have mut-p53, their *SLC7A11* expression is much lower compared to those cells with wt-p53. Therefore, ESCC cells with mut-p53 showed enhanced cytotoxic sensitivity to oridonin. To interrogate the functional relationship between *SLC7A11* and cellular sensitivity to oridonin, we knocked down *SLC7A11* in EC109 cells. We found that inhibition of *SLC7A11* expression significantly increased the sensitivity of EC109 cells to oridonin and intracellular ROS. Finally, to further explain the effect of intracellular GSH content on the sensitivity of oridonin, we tested their effects on cell viability, GSH and ROS levels by the combined application of oridonin or APR-246, and BSO. We found that the use of oridonin alone, APR-246 or BSO were less toxic to 109 cells, but after combined use, it significantly increased cytotoxicity, and the combined effect of oridonin was significantly stronger than APR-246.

In summary, this study validates our hypothesis that GSH acts as a target for oridonin. Esophageal squamous carcinoma cells with lower expression of *SLC7A11* and mut-p53 are more sensitive to oridonin and are more susceptible to oxidative stress. For esophageal cancer cells with a high level of *SLC7A11*, GSH can be synergistically depleted by the combined use of oridonin and γ-glutamyl cysteine synthetase inhibitor (BSO), leading to irresistible ROS accumulation and cell death. Therefore, we can formulate a reasonable plan for the clinical treatment of oridonin according to the expression of *SLC7A11* and mut-p53.

## Data Availability Statement

The datasets generated for this study can be found in The National Omics Data Encyclopedia (NODE). Data link: https://www.biosino.org/node (Accession No: OEP000514).

## Author Contributions

QZ and YG designed, performed the research, and wrote the paper. YL, NL, and TA did all the experiments and helped to write the paper. JS did the RNA-seq analysis. HL, JG, and WT helped with the discussion and design of the paper.

### Conflict of Interest

The authors declare that the research was conducted in the absence of any commercial or financial relationships that could be construed as a potential conflict of interest.
