# Supplementary material for: Involvement of Glutathione Depletion in Selective Cytotoxicity of Oridonin to p53-Mutant Esophageal Squamous Carcinoma Cells
Source: Front Oncol. 2020 Jan 15;9:1525. doi: 10.3389/fonc.2019.01525 (PMC6974803; doi:10.3389/fonc.2019.01525)
Supplement: Supplementary file 1 [file Table_1.DOCX]

**Supplementary Table 1.** A list of the top up- and down-regulated genes (arranged by log2 fold change) are shown, in TE1 cells treated with 30 μM oridonin for 4 hr. Data was analysed using RNA-sequencing.

| **Gene ID** | **geneName** | **baseMean** | **Log Fold Change** | **P.Value** |
| --- | --- | --- | --- | --- |
| ENSG00000173110 | HSPA6 | 4214 | 5.33 | 0.00E+00 |
| ENSG00000204388 | HSPA1B | 117216 | 4.74 | 0.00E+00 |
| ENSG00000125740 | FOSB | 3268 | 4.63 | 0.00E+00 |
| ENSG00000204389 | HSPA1A | 172813 | 4.51 | 0.00E+00 |
| ENSG00000170345 | FOS | 4813 | 3.89 | 0.00E+00 |
| ENSG00000087074 | PPP1R15A | 14725 | 3.77 | 0.00E+00 |
| ENSG00000186594 | MIR22HG | 1915 | 3.71 | 0.00E+00 |
| ENSG00000099860 | GADD45B | 5085 | 3.69 | 0.00E+00 |
| ENSG00000132002 | DNAJB1 | 18113 | 3.51 | 0.00E+00 |
| ENSG00000130766 | SESN2 | 2304 | 3.44 | 0.00E+00 |
| ENSG00000113070 | HBEGF | 1321 | 3.38 | 6.18E-278 |
| ENSG00000120738 | EGR1 | 16966 | 3.32 | 0.00E+00 |
| ENSG00000178381 | ZFAND2A | 2719 | 3.09 | 0.00E+00 |
| ENSG00000151929 | BAG3 | 7767 | 3.04 | 0.00E+00 |
| ENSG00000100292 | HMOX1 | 2051 | 2.89 | 5.63E-283 |
| ENSG00000150991 | UBC | 85116 | 2.74 | 0.00E+00 |
| ENSG00000116285 | ERRFI1 | 7553 | 2.29 | 0.00E+00 |
| ENSG00000162783 | IER5 | 7485 | 2.26 | 0.00E+00 |
| ENSG00000120694 | HSPH1 | 9042 | 1.83 | 6.02E-263 |
| ENSG00000143384 | MCL1 | 7893 | 1.69 | 0.00E+00 |
| ENSG00000117289 | TXNIP | 845 | -3.044 | 8.18E-190 |
| ENSG00000179431 | FJX1 | 204 | -2.421 | 2.48E-42 |
| ENSG00000177494 | ZBED2 | 214 | -2.113 | 2.09E-37 |
| ENSG00000177971 | IMP3 | 412 | -2.036 | 2.09E-51 |
| ENSG00000168040 | FADD | 510 | -1.993 | 1.71E-62 |
| ENSG00000023445 | BIRC3 | 662 | -1.926 | 1.53E-92 |
| ENSG00000169515 | CCDC8 | 352 | -1.718 | 2.37E-38 |
| ENSG00000179041 | RRS1 | 472 | -1.444 | 3.84E-39 |
| ENSG00000153395 | LPCAT1 | 1194 | -1.44 | 1.66E-72 |
| ENSG00000182704 | TSKU | 968 | -1.252 | 5.18E-47 |
| ENSG00000272620 | AFAP1-AS1 | 596 | -1.248 | 4.21E-38 |
| ENSG00000107984 | DKK1 | 1619 | -1.173 | 1.61E-64 |
| ENSG00000167173 | C15orf39 | 1184 | -1.135 | 2.67E-46 |
| ENSG00000186834 | HEXIM1 | 1430 | -1.125 | 4.80E-60 |
| ENSG00000215012 | C22orf29 | 1514 | -1.069 | 1.89E-40 |
| ENSG00000162613 | FUBP1 | 1895 | -1.028 | 2.64E-54 |
| ENSG00000196700 | ZNF512B | 1141 | -1.011 | 3.41E-41 |
| ENSG00000183741 | CBX6 | 3267 | -0.61 | 1.09E-35 |
| ENSG00000135486 | HNRNPA1 | 10204 | -0.523 | 1.23E-37 |
| ENSG00000058085 | LAMC2 | 23636 | -0.444 | 4.48E-39 |

**Supplementary Table 2.** A list of the top up- and down-regulated genes (arranged by log2 fold change) are shown, in EC109 cells treated with 30 μM oridonin for 4 hr. Data was analysed using RNA-sequencing

| **Gene ID** | **geneName** | **baseMean** | **Log Fold Change** | **P.Value** |
| --- | --- | --- | --- | --- |
| ENSG00000204389 | HSPA1A | 52563 | 1.899 | 0.00E+00 |
| ENSG00000204388 | HSPA1B | 37968 | 1.653 | 3.68E-179 |
| ENSG00000151012 | SLC7A11 | 8956 | 1.239 | 8.75E-68 |
| ENSG00000140961 | OSGIN1 | 1100 | 1.175 | 4.78E-53 |
| ENSG00000186594 | MIR22HG | 443 | 1.116 | 5.44E-21 |
| ENSG0000015192 | BAG3 | 3995 | 0.925 | 4.92E-52 |
| ENSG00000091592 | NLRP1 | 972 | 0.885 | 1.82E-25 |
| ENSG00000023909 | GCLM | 4579 | 0.824 | 1.70E-51 |
| ENSG00000059145 | UNKL | 1731 | 0.815 | 2.61E-32 |
| ENSG00000198431 | TXNRD1 | 30545 | 0.804 | 1.74E-83 |
| ENSG00000132002 | DNAJB1 | 11115 | 0.778 | 6.48E-33 |
| ENSG00000120694 | HSPH1 | 18492 | 0.727 | 2.81E-39 |
| ENSG00000108448 | TRIM16L | 3556 | 0.662 | 8.38E-28 |
| ENSG00000271303 | SRXN1 | 6339 | 0.63 | 6.92E-22 |
| ENSG00000115641 | FHL2 | 3659 | 0.604 | 2.07E-24 |
| ENSG00000221926 | TRIM16 | 3174 | 0.596 | 1.43E-23 |
| ENSG00000145623 | OSMR | 9525 | 0.543 | 5.49E-20 |
| ENSG00000110172 | CHORDC1 | 16201 | 0.531 | 2.62E-25 |
| ENSG00000106105 | GARS | 8226 | 0.496 | 1.41E-20 |
| ENSG00000109971 | HSPA8 | 182652 | 0.487 | 1.68E-30 |
| ENSG00000166963 | MAP1A | 413 | -0.915 | 5.10E-15 |
| ENSG00000134215 | VAV3 | 177 | -0.869 | 1.18E-09 |
| ENSG00000114790 | ARHGEF26 | 272 | -0.837 | 1.25E-10 |
| ENSG00000188158 | NHS | 869 | -0.786 | 1.37E-10 |
| ENSG00000165300 | SLITRK5 | 349 | -0.778 | 3.38E-10 |
| ENSG00000105641 | SLC5A5 | 496 | -0.721 | 3.48E-11 |
| ENSG00000157657 | ZNF618 | 679 | -0.608 | 3.66E-09 |
| ENSG00000183251 | OR51B4 | 1194 | -0.523 | 5.23E-10 |
| ENSG00000149418 | ST14 | 1664 | -0.507 | 1.47E-12 |
| ENSG00000001167 | NFYA | 1585 | -0.497 | 1.32E-10 |
| ENSG00000185551 | NR2F2 | 3446 | -0.496 | 4.39E-15 |
| ENSG00000186205 | 1-Mar | 3824 | -0.495 | 1.96E-20 |
| ENSG00000072422 | RHOBTB1 | 2287 | -0.47 | 4.48E-11 |
| ENSG00000244462 | RBM12 | 6010 | -0.457 | 4.50E-13 |
| ENSG00000118246 | FASTKD2 | 1844 | -0.455 | 6.90E-10 |
| ENSG00000189060 | H1F0 | 2931 | -0.447 | 2.28E-10 |
| ENSG00000041353 | RAB27B | 1713 | -0.434 | 9.59E-10 |
| ENSG00000153395 | LPCAT1 | 11862 | -0.416 | 2.71E-18 |
| ENSG00000137310 | TCF19 | 3480 | -0.351 | 3.66E-09 |
| ENSG00000162636 | FAM102B | 4502 | -0.348 | 2.12E-09 |
